# Multicomponent physical activity program to prevent body changes and metabolic disturbances associated with antiretroviral therapy and improve quality of life of people living with HIV: a pragmatic trial

**DOI:** 10.6061/clinics/2021/e2457

**Published:** 2021-03-15

**Authors:** Elisabete Cristina Morandi dos Santos, Alex Antonio Florindo, Ardiles Vitor Santos, Camila de Melo Picone, Túlio Gamio Dias, Aluisio Cotrim Segurado

**Affiliations:** I Divisao/Departamento de Molestias Infecciosas e Parasitarias, Hospital das Clinicas HCFMUSP, Faculdade de Medicina, Universidade de Sao Paulo, Sao Paulo, SP, BR.; II Escola de Ciencias, Artes e Humanidades, Universidade de Sao Paulo, Sao Paulo, SP, BR.; III Prefeitura Municipal de Joinville, Joinville, SC, BR.

**Keywords:** HIV, Physical Activity, Primary Prevention, Lipodystrophy, Quality of Life

## Abstract

**OBJECTIVES:**

Comprehensive care for people living with human immunodeficiency virus (HIV) (PLH) includes the promotion of healthier habits, including physical activity (PA). This study aimed to describe a multicomponent pragmatic trial protocol to assess the effect of PA in preventing body changes and metabolic disturbances, improving the quality of life of PLH starting antiretroviral therapy (ART) and present cohort characteristics.

**METHODS:**

PLH undergoing ART for ≤4 months were recruited for a randomized trial. The intervention comprised three cardiorespiratory and/or strength training sessions per week at the clinic or in public spaces for 6 months under on-site or remote supervision, and educational sessions. Participants’ PA levels, cardiorespiratory fitness, anthropometric measures, strength, flexibility, quality of life, and laboratory monitoring (blood glucose and lipids, CD4 counts) at baseline and post-intervention will be compared. The pragmatic design aims to enable the assessment of intervention effectiveness in real-life conditions.

**RESULTS:**

At baseline, our cohort of 38 recently diagnosed patients (mean time since HIV diagnosis and duration of ART were 3 and 2.58 months, respectively) were predominantly male, young, with high schooling and good immune status (median CD4 count=498 cells/mm^3^). Twenty-two (57.9%) patients reported a PA below the World Health Organization recommendations. We found baseline normal anthropometric measures and metabolic parameters: below-average trunk flexion and elbow extension strength, poor handgrip strength and flexibility, and high quality of life scores in all except the physical domain.

**CONCLUSIONS:**

Understanding how effective PA is in preventing body changes and metabolic disturbances, and in improving the quality of PLH starting ART may help establish guidelines to better incorporate PA in HIV care.

## INTRODUCTION

Combined antiretroviral therapy (ART) has resulted in remarkable changes in the human immunodeficiency virus/ acquired immunodeficiency syndrome (HIV/AIDS) pandemic, by reducing the HIV-related morbidity and mortality globally and contributing to decreased viral transmission (1). In Brazil, universal access to ART has been provided free of charge by the public health system since 1997, and in 2013, it was scaled-up with the “test-and-treat” strategy that recommends ART to people living with HIV (PLH) regardless of their immune status (2). In this context, with a significant reduction in the incidence of opportunistic infections and increased survival of PLH, specialized services are faced with novel healthcare needs associated with chronic HIV infection, including lipodystrophy (body changes and metabolic disturbances) and various organ dysfunctions (cardiovascular, kidney, bone, and/or liver) that significantly impair patients’ quality of life (3).

The changes in the body associated with lipodystrophy include peripheral lipoatrophy (face, limbs, and buttocks) and fat accumulation in the neck, chest, breasts, back, and abdomen (4). The prevalence of such changes varies significantly among cohorts, depending on the duration of the HIV infection and ART, exposure to different antiretrovirals, dietary habits, and the history of physical activity (PA), including exercise (5). Moreover, body changes when perceived by family members, friends, and coworkers can lead to stigma and damage the patients’ social relationships (6,7), which is one of the quality-of-life domains. Among 365 PLH evaluated in São Paulo, for instance, lower scores were observed in the social relationships and environment domains of quality of life, as compared to the physical and psychological domains (8).

In addition, chronic HIV infection is associated with peripheral insulin resistance and diabetes, as well as dyslipidemia, characterized by hypertriglyceridemia, reduced HDL-cholesterol, and elevated LDL-cholesterol levels (9). Furthermore, elevated blood concentrations of inflammatory biomarkers, such as IL-6, IL-8, IL-15, and TNF-α, have been reported in PLH (9- 10). Altogether, chronic immune activation, inflammation, and metabolic alterations are believed to contribute to the development of age-related diseases (liver dysfunction, neurological decline) and atherosclerosis in PLH, ultimately leading to enhanced cardiovascular risk (10,11).

Scientific evidence supports the benefit of PA, which may include exercise which helps in preventing obesity, cardiovascular diseases, the onset of type 2 diabetes and osteoporosis, and improving the overall well-being and quality of life in the general population (12). As far as PLH are concerned, the scores of leisure-time PA were shown to be inversely associated with central fat indicators, such as the waist-hip ratio and the sum of skinfolds in the central body in a cross-sectional study of 220 patients followed up at an HIV reference center in São Paulo (13). Likewise, intervention studies involving PLH have demonstrated that exercise, a component of PA, improves muscular strength and cardiorespiratory fitness and contributes significantly to reducing the central fat (both visceral and subcutaneous), enhances the quality of life (14,15), and leads to normal blood lipid levels (16). Moreover, systematic reviews and meta-analysis studies have concluded that PA is safe and beneficial for PLH, as it helps improve cardiorespiratory fitness and muscular strength (17,18). However, concerns have been raised about the difficulty in ensuring adherence to PA interventions among these patients during the follow-up (17).

Regardless of the efficacy of PA in improving the body composition and reverting metabolic abnormalities, the extent to which PA can prevent lipodystrophy and other adverse effects of chronic HIV infection and ART remains unclear. It is notable that the studies published so far have not been properly designed to answer this question. Therefore, the present study aimed to evaluate the effectiveness of a structured multicomponent PA program in preventing body changes and metabolic disturbances and in improving the quality of life of PLH that are undergoing ART.

For this intervention, we adopted a pragmatic design, establishing a protocol to be implemented close to participants’ real-life conditions. Given the spread of HIV infection among the less privileged segments of society in Brazil (19), it is necessary to reduce the barriers to interventions and propose alternative models that may be more acceptable and feasible in the everyday life of PLH to maximize the patients’ enrollment and adherence. One of these possibilities includes offering home-based interventions to be carried out at home or as close as possible to where patients live, including surrounding parks, squares, open-air gyms, or other public spaces. Using a combined training and educational approach, we aimed to empower PLH to autonomously make the best use of locally available infrastructure to engage in PA.

## METHODS

This pragmatic multicomponent randomized trial was carried out at the HIV clinic (SEAP), an outpatient care setting affiliated with the Division of Infectious Diseases, Hospital das Clinicas, Faculty of Medicine, University of São Paulo. The eligible subjects included patients with confirmed HIV infection, following diagnostic guidelines issued by the Brazilian Ministry of Health, aged 18 to 59 years, who were undergoing ART for no longer than 4 months. As our main goal was to investigate the effect of PA in preventing body changes and metabolic disturbances associated with ART, the patients who exhibited such manifestations *a priori* were not admitted to the study. Therefore, we excluded patients with a waist circumference >102 cm for men or 88 cm for women, those with fasting blood glucose ≥100 mg/dL, total blood cholesterol ≥239 mg/dL, LDL-cholesterol ≥160 mg/dL, or triglyceride level ≥150 mg/dL. Additionally, the patients diagnosed with uncontrolled hypertension, stroke, cancer, or pregnant women; those taking anabolic steroids, hypolipidemic, or hypoglycemic medications; those who reported aesthetic surgery (lipoaspiration, liposculpture, or any other plastic surgery) in the previous 12 months and those who had contraindications for PA, (for instance, PLH whose health status might worsen with the intervention, such as patients with severe musculoskeletal conditions and highly immunocompromised individuals) were excluded.

### Subject enrolment and cohort characteristics

Using consecutive sampling, we recruited patients who attended the clinic for a consultation with an infectious disease physician. The patients were screened for inclusion and exclusion criteria and were invited to enroll in the study by a team of trained interviewers with a college degree in the health area. At the same time, as part of the pre-intervention assessment, the patients underwent a face-to-face interview with the research team for collection of socio-demographic data, and information about smoking and alcohol consumption.

### Experimental design protocol

After admission to the study, the participants were randomly allocated in blocks of six to the intervention and control groups at a 1:1 ratio, using the EPI INFO software module Random Number List Generator. The randomization procedure was carried out by a member of the research team blinded to the study. The subjects allocated to the intervention group will undergo a physical training program, supervised on-site and/or remotely, and participate in educational sessions to promote PA.

The training program consists of three 60-minute sessions per week, two with cardiovascular and strength exercises, and one with cardiorespiratory training only, including warm-up, steady state workload, and stretching/resting, for 6 months, to be conducted at the clinic or in public spaces (involving walking, running, and bicycling, and the use of elastic bands and available exercise equipment), with on-site and/or remote supervision by a physical education professional, using a home-based strategy. The patients who might not attend all face-to-face sessions will be counseled to incorporate the same training program in their daily routine, and those who already exercise will be asked to incorporate the cardiorespiratory or strength components, or to maintain or enhance their regular PA, according to their needs and possibilities. The training program takes into account the individual history of PA, health record, results obtained in the cardiorespiratory fitness and strength assessment, following the guidelines from the World Health Organization (20). After scheduling the first PA session, all participants will receive at least three Facebook/Whatsapp/SMS messages every week, encouraging texts/videos/website links, incentive apps, and updates about the PA.

### Strength training session

The strength training consists of 25-30-minute sessions, using equipment available in public spaces (open-air gyms, bleachers, and stairs) and elastic bands provided for the study. The program will follow a standardized protocol that includes 1) extension of the knee, 2) flexion of the knee, 3) ankle dorsiflexion, 4) horizontal flexion of the shoulders and scapular abduction, 5) scapular adduction and inferior scapular rotation, 6) ulnar extension, 7) ulnar flexion, and 8) forward flexion of the trunk. Strength exercises (7 to 8) will be performed in multiple series as follows: three series of 10 repetitions for individuals with prior experience in regular exercise or one series of 15 repetitions for beginners (24). Repetitions will be performed at moderate speed, followed by a 1-to 3-minute passive break between series. The intensity of training will be gradually increased, starting at 70%, and is limited to 80% of maximal repetition (1-RM) (21). As such, untrained subjects will be kept on a single series with 15 repetitions for 2 to 4 weeks, with an estimated intensity between 60 and 70% of 1-RM. In contrast, those with regular exercise practice will start training with three series of 10 repetitions, with an estimated intensity of 70-80% of 1-RM for 2 months. The OMNI Subjective Effort Scale (22) will be used to estimate the intensity of the strength training. According to this tool, each participant will estimate the difficulty of the effort for themselves, using a scale that ranges from zero (extremely easy) to 10 (extremely hard). Based on this estimate, a specific color band will be chosen to keep the exercise effort between 5 and 7 points on the OMNI scale (22). Each strength training session will be followed by 7 to 10 minutes of stretching.

### Cardiorespiratory training session

The cardiorespiratory training involves 30-minute sessions of indoor and outdoor walking and running exercises (21), in settings such as bleachers, stairs, or walking and running lanes in public spaces, at an intensity of 70-85% of maximal heart rate. To monitor the intensity of training, portable heart rate monitoring devices and the Borg Subjective Effort Scale will be used (23).

The strength and cardiorespiratory training sessions will be supervised on-site, when carried out at the HIV clinic, or remotely, using phone calls or text messages, when performed in other locations in the home-based strategy.

### Educational intervention session

Group face-to-face educational sessions will be held monthly with participants allocated to the intervention group to promote PA, wherein guidance on the use of alternative equipment at home or in nearby locations (parks, squares, clubs, open-air gyms, etc.) will be provided, activities that may be practiced, and the potential barriers to adopting a healthier lifestyle will be discussed.

### Adherence to the intervention

The participants' adherence to the experimental protocol will be reinforced using telephone calls or SMS messages, according to the participant’s record of on-site supervised exercise or feedback report on remote activities.

### Study outcomes

The study outcomes include self-reported current PA levels using the long version of the International Physical Activity Questionnaire (IPAQ) with the assessment of PA related to leisure-time mobility (walking and bicycling), moderate-intensity (MPA) and vigorous PA (VPA) in sports and physical exercises; objective current PA level (accelerometer); cardiorespiratory fitness (ergospirometry); anthropometric measures (body mass index [BMI] and waist-hip ratio); strength (hand grip, trunk flexion - sit-up, and elbow extension - push-up tests), and flexibility (Wells’ bench test), quality of life (using the WHOQOL-bref questionnaire (24), and laboratory monitoring (fasting blood glucose, total blood cholesterol, HDL-cholesterol, LDL-cholesterol, triglycerides, and CD4 counts).

### Data analysis

Among the study variables of interest, the categorical data are presented as absolute numbers and frequencies, and numerical data, using central tendency and dispersion measures (mean, median, standard deviations, and interquartile range, as appropriate). The Kolmogorov-Smirnov test was used to assess the variable distribution in the cohort. In this paper, we describe the cohort characteristics and baseline results of study outcomes. After the completion of the protocol, the effect of the intervention will be assessed using the hypothesis of non-modification of the experimental group over time (T0 [baseline] *versus* T1 [post-intervention]) compared to the control group by means of a non-parametric repeated measures ANOVA model. The analyses will be conducted with the support of the following software: R nparLD package (https://www.r-project.org/), IBM SPSS 21 (Statistical Package for the Social Sciences), and Excel 2010® (Microsoft Office), using a significance level of 0.05. Randomization was expected to minimize the selection bias.

### Ethical issues

The study was conducted in accordance with the national recommendations for research involving human subjects. Participation was voluntary, and the inclusion followed informed consent. The research protocol was approved by the Institutional Review Board (CAPPesq), Hospital das Clínicas, Faculdade de Medicina, Universidade de São Paulo. Moreover, the participants allocated to the control group received guidance on the benefit of PA and were invited to attend face-to-face supervised PA sessions after the study completion.

## RESULTS

### Subject recruitment and cohort characterization

**Figure 1:**
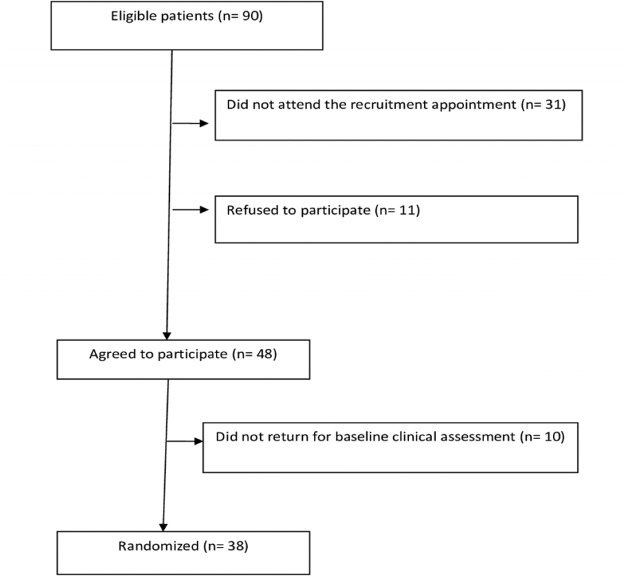
Recruitment and selection of patients admitted to the study. SEAP, 2014-6.

Of the 90 eligible patients, 31 did not attend the recruitment appointment. An additional 11 individuals refused to participate, and 10 did not return for baseline clinical assessment. Thus, our cohort comprised 38 patients with PLH starting ART (Figure 1).

1. Socio-demographic characteristics
  The majority of the participants were men (87%), single (73.7%), and self-reported as black or mulatto (65.8%). They were aged between 19 and 55 years (mean=32.6; SD=9.08) and more often lived in the city of São Paulo (68.4%). The mean schooling of the participants was 11.9 years and family income varied between 750 and 20,000 reais (median=3,000). The majority of the participants (57.9%) reported drinking at least one alcoholic drink at least once or twice a week, and 34.2% were current smokers (Table 1).
2. Physical Activity - self-reported current PA
  The self-reported PA a week prior to inclusion in the study, assessed using the IPAQ questionnaire, revealed a total median reported time of moderate and vigorous-intensity PA (MVPA) during the leisure-time, including recreation, sports, and exercise of zero minutes/week (IQR=0-370). Likewise, a median time of zero minutes/week (IQR=0-24) of activity was reported for leisure-time walking. Based on their self-report of PA, 22 (57.9%) patients in our cohort did not meet the World Health Organization (WHO) recommendations of at least 150 minutes per week of MVPA (25).
3. Accelerometry
  The objective assessment of PA in the week prior to the inclusion in the study was successfully performed in 30 (78.9%) subjects using an accelerometer. Among these, 28 participants (93.3%) practiced moderate-intensity PA (MPA), although none engaged in VPA. Based on their accelerometer records, 20 (66.6%) patients met the WHO recommendations of at least 150 minutes per week of MVPA (25) (Table 2).
4. Cardiorespiratory fitness
  Regarding cardiorespiratory fitness, most (83.3%) participants had a maximum VO_2_ value analyzed by ergospirometry that could be classified as good or excellent for their age and gender, according to the American College of Sports Medicine standards (26), with a mean result of 45.75 mL/kg/minute.
5. Physical assessment
  We found normal anthropometric measures (BMI and WHR) in our cohort. In contrast, the patients’ physical assessment revealed below-average results for the sit-up (trunk flexion) and push-up (elbow extension) strength tests, and poor hand grip strength and flexibility (Table 3).
6. Quality of Life
  The scores obtained using the WHOQoLHIV Bref questionnaire were high for most quality-of-life domains, being classified in the fourth quartile for the psychological, independence level, social relations, environment, and spirituality/religion/personal belief domains. However, the scores for the physical domain were within the intermediate classification range (Table 4).
7. Laboratory assessment of immunological status and metabolic parameters
  The patients in our cohort presented with a good immunological status (median CD4 count=498 cells/mm^3^). Likewise, blood glucose and lipid concentrations were within the normal range (Table 5).

## DISCUSSION

To our knowledge, this is the first clinical trial designed to evaluate the effectiveness of PA in preventing body changes and metabolic disturbances associated with HIV infection and ART. Previous intervention studies have addressed the impact of PA in PLH, including those who already presented with body changes or metabolic disturbances (11-13). Furthermore, our approach has been designed as a multicomponent intervention program, including cardiorespiratory and strength training, in parallel with educational sessions to promote the PA.

Considering the adoption of the “test-and-treat” strategy” by the Brazilian Ministry of Health in 2013, which recommends ART for all PLH regardless of the immune status, the intervention we propose is relevant to provide evidence to support the incorporation of PA early in the comprehensive care of PLH. In addition, our clinical trial was conceived using a pragmatic design to allow the experimental intervention to take place in real-life conditions of PLH followed up at a reference outpatient clinic. In contrast to explanatory trials, which are usually designed to confirm a physiological or clinical hypothesis based on strictly controlled interventions (27), in pragmatic trials, additional emphasis is provided to the external validity of the study, that is, the ability to generalize results to provide evidence for the adoption of new clinical protocols in real-world practice. In this scenario, the effectiveness of the tested intervention was scrutinized. The proposal of more feasible interventions helps to reduce the barriers to the access and enhance the adherence of patients in real life. Despite being recognized as an international pioneer in HIV care and ART policies as universal access to medication is guaranteed free of charge within the public health system, Brazil faces striking and challenging social inequalities that may hinder the overall uptake of PA recommendations by the most vulnerable PLH.

The socio-demographic profile of our cohort is concordant with the epidemiological characteristics of the current HIV epidemic in São Paulo. In fact, the most recently diagnosed PLH and, therefore, most patients who are being started on ART in the city are young men (28). In terms of the educational background, our data reemphasize previous reports that show that the patients under follow-up at SEAP usually have high schooling (29). Moreover, they presented with good clinical and immunological status, as expected in early HIV diagnosis.

Another important finding of our study is the observation that one-third of the cohort consisted of current smokers, in contrast to evidence from population surveys that show smoking has recently become less frequent among young Brazilian men (30). Indeed, high current smoking rates have been previously reported in other Brazilian and foreign PLH cohorts (31 [Bibr B32] 33), sometimes two-to three-fold higher than those found in the general population. This increased risk is believed to be associated with emotional distress, anxiety, and depression (32,33). Thus, we highlight the need to incorporate smoking cessation strategies as a primary focus when establishing the guidelines to promote healthier lifestyles for PLH being started on ART.

In terms of the involvement in PA, the population admitted to the study can be considered as insufficiently active, due to the median reported time of leisure-time MVPA of zero minutes/week (IQR 0-370) and zero minutes/week (IQR=0-24) spent walking. Only 16 (42.1%) patients reported practicing more than 150 minutes of MVPA per week, as recommended by the World Health Organization in 2010 (25). However, the use of an objective PA assessment method (accelerometry) showed that a larger proportion of subjects (20/30, 66.6%) exceeded the same PA recommendations. This underestimation has been previously reported with IPAQ compared to accelerometry (34). In our study, it is likely due to the fact that we only assessed leisure-time PA. Moreover, the questionnaire requires the respondents to report only activities that are practiced for 10 minutes or longer; therefore, activities of shorter duration might have been missed. Regardless of the method used to assess PA activity (self-report *versus* objective measurement), given that the WHO updated its recommendations for at least 150-300 minutes of PA per week in 2020 (23), the need to promote PA among PLH in our cohort is further reinforced.

Regardless of the observed low engagement in PA, our patients’ cardiorespiratory fitness at admission to the study was classified as good or excellent, according to the American College of Sports Medicine standards (26). The deterioration of such conditions is known to be associated with HIV disease progression when immunological and clinical compromise may lead to changes in energy metabolism, reducing the patients’ resistance to fatigue and impairing ventilatory mechanics (35). It should be noted that our cohort consisted of recently diagnosed PLH, with an overall good immunological status. In this context, they were observed to perform well from the point of view of cardiorespiratory fitness.

The limitations of this study should be considered. The use of stringent inclusion and exclusion criteria led to a reduced number of eligible subjects, particularly because enrolment occurred in a single HIV clinical setting. As such, a broad generalization of the intervention effect may not be possible.

Nevertheless, we expect the proposed intervention strategy to allow PLH to engage in PA, including exercises at the HIV outpatient clinic, at home or in other nearby locations, using locally available low-cost infrastructure, under on-site or remote supervision. This flexible protocol aims to increase the adherence to the intervention and provide evidence of the effect of PA in preventing the body changes and metabolic disturbances associated with HIV infection and ART, and to improve the quality of life of PLH.

A better understanding of the effectiveness of this multicomponent intervention will certainly be useful to support the incorporation of PA in HIV comprehensive care within the Brazilian public health system. Additionally, this approach may contribute to empowering PLH to make the best use of resources available in services or in the community, facilitating engagement in PA as part of their daily lives.

## AUTHOR CONTRIBUTIONS

Santos ECM was responsible for the study conception, data collection, analysis and interpretation, manuscript writing and critical review. Florindo AA was responsible for the study conception, data interpretation, and manuscript writing and critical review. Picone CM, Santos AV and Dias TG were responsible for the data collection and interpretation, and manuscript critical review. Segurado AC was responsible for the study conception, data analysis and interpretation, and manuscript writing and critical review.

## Figures and Tables

**Table 1:** Socio-demographic characteristics. SEAP, São Paulo, 2014-6.

Characteristic	N	%
Sex		
male	33	86.8
female	5	13.2
Self-reported skin color		
white	13	34.2
mixed (mulatto)	19	50.0
black	6	15.8
Place of birth		
São Paulo state	15	39.5
other states	23	60.5
Residence		
São Paulo city	26	68.4
elsewhere	12	31.6
Occupation		
employed	33	86.8
student	1	2.6
unpaid leave/unemployed	4	10.6
Marital status		
single	28	73.7
married	5	13.1
living together	2	5.3
separated/divorced	3	7.9
Current smoking		
yes	13	34.2
no	25	65.8
Alcohol consumption		
1-2/week	22	57.9
3-4/week	1	2.6
never	15	39.5

**Table 2:** Physical Activity level (minutes/week) in the previous week-accelerometer. SEAP, São Paulo, 2014-6.

Type of physical activity*	Minutes/weekMedian (IQR)#
Sedentary behavior	2,929 (2,185-3383)
Light-intensity physical activity (LPA)	1,410 (1,191-1858)
Moderate-intensity physical activity (MPA)	286 (176-411)
Total number of steps/week (LPA+MPA+VPA)	52,153 (36,300-69,155)

* Valid data (≥3 days of use, one d on the weekend, for ≥6h/day) for 30 patients.

# Counts were converted into minutes per week (total time the patient remains with such counts).

**Table 3:** Physical evaluation. SEAP, São Paulo, 2014-6.

Variable	Median (IQR)
BMI	23.22 (22.23-26.03)
WHR	0.83 (0.79-0.88)
Hand grip strength test (kg)	76.50 (67.62-87.25)
Trunk flexion strength test (repetitions/minute)^#^	20.50 (12.00-26.50)
Elbow extension strength test (total repetitions)*	15.00 (9.25-19.75)
Flexibility test (range in cm)*	21.25 (13.91-26.35)

BMI, body mass index; WHR, waist-hip ratio; *Missing data for two patients; ^#^Missing data for four patients.

**Table 4:** Quality-of-Life scores. SEAP, São Paulo, 2014-6.

Domain	Median (IQR)^#^
Physical	11.73 (10.75-14.00)
Psychological	15.20 (12.80-17.00)
Independence level*	15.00 (12.00-17.00)
Social relations	15.00 (12.75-17.00)
Environment	13.50 (12.37-16.00)
Spirituality/religion/personal beliefs	15.50 (11.00-18.00)

^#^Scores for each domain ranged from four to 20. *Missing data for one patient.

**Table 5:** Laboratory monitoring data. SEAP, São Paulo, 2014-6.

Variable	Median (IQR)
CD4 count (cells/mm^3^)	498 (344-645)
HDL-cholesterol (mg/dL)^*^	44.00 (38.00-52.00)
LDL-cholesterol (mg/dL)^*^	98.00 (78.00-113.50)
Triglycerides (mg/dL)^#^	96.50 (78.50-124.25)
Fasting blood glucose (mg/dL)	86.00 (78.50-89.75)

*Missing data for one patient; ^#^Missing data for two patients.
